# The metabolic basis of adolescent idiopathic scoliosis: 2011 report of the “metabolic” workgroup of the Fondation Yves Cotrel

**DOI:** 10.1007/s00586-012-2245-8

**Published:** 2012-03-09

**Authors:** Emre Acaroglu, Regis Bobe, Jocelyn Enouf, Ralph Marcucio, Florina Moldovan, Alain Moreau

**Affiliations:** 1 Ankara Spine Center, Iran caddesi 45/2, Kavaklidere, Ankara, 06700 Turkey; 2 U689 INSERM, Hôpital Lariboisière, Paris, France; 3 Department of Orthopedic Surgery, University of California, San Francisco, California, USA; 4 Department of Stomatology, Faculty of Dentistry, CHU Sainte Justine, Université de Montréal, Montreal, QC Canada; 5 Viscogliosi Laboratory in Molecular Genetics of Musculoskeletal Diseases, Sainte-Justine University Hospital Research Center, Montreal, QC Canada; 6 Department of Stomatology, Faculty of Dentistry, Université de Montréal, Montreal, QC Canada; 7 Department of Biochemistry and Department of Surgery, Faculty of Medicine, Université de Montréal, Montreal, QC Canada

**Keywords:** Adolescent idiopathic scoliosis, Melatonin, Calmodulin, Estrogen, Bipedality

## Abstract

**Objective:**

The purpose of this review is to elucidate the metabolic processes involved in the pathogenesis of adolescent idiopathic scoliosis (AIS) in light of research by the present authors as well as current literature.

**Methods:**

Pathogenetic mechanisms involved in AIS were modeled as (a) a form of neuromuscular scoliosis (in conjunction with an adverse mechanical environment such as bipedality), in which hormonal and other chemical factors act as regulators of skeletal muscle tone and function; (b) as a consequence of an abnormality in growth of the spinal column (in conjunction with an adverse mechanical environment such as bipedality), in which hormones and other chemical factors act as regulators of growth; and (c) as a mechanical failure of one side of the vertebral column due to a defect in trabecular formation or mineralization (in conjunction with an adverse mechanical environment such as bipedality); in which hormonal and other chemical factors act as regulators of bone formation, mineralization and/or resorption.

**Results and conclusion:**

Current evidence supporting these models individually or in combination is discussed.

## The etiology of adolescent “idiopathic” scoliosis remains unresolved

Idiopathic scoliosis is a deformity of the torso that involves all three planes of the body and is associated with lateral deviation and axial rotation of the involved segments as well as substantial lordosis if located in the thoracic spine. Infantile and juvenile/adolescent idiopathic scoliosis (AIS) need to be distinguished, because they are likely to be distinct entities. Henceforward, the term ‘idiopathic” scoliosis is going to be used to define specifically AIS.

The search for an etiology of AIS may sound like an oxymoron. However, to the best of our knowledge, idiopathic scoliosis is probably not as “idiopathic” as previously considered. Starting in the late 1960s, substantial research efforts have been directed to investigate possible mechanisms, none of which explain all the different facets of this complex disease. A detailed overview of this previous work is going to be included here only briefly, as it has been reviewed in detail in several recent articles [1–5].

There is substantial evidence that there is a familial/genetic background, but genetic studies have failed to identify a single genetic locus or even a single chromosome that contributes to AIS [3, 6]. These results may indicate that AIS has a multifactorial etiology. Several theories and/or factors have been proposed to explain the pathogenesis of AIS, including connective tissue disorders, skeletal muscle/contractile tissue disorders, hormonal perturbations, developmental imbalance, abnormal vestibular and proprioceptive systems, aberrant biomechanical factors, uncoupled neuro-osseous growth, and dissociation between the timing of skeletal and CNS maturation. Most, if not all of these factors and conditions are likely present in individuals with AIS, and may either result from AIS, or participate in disease onset and/or progression. Thus, distinguishing the primary underlying pathogenetic factor(s) from the secondary and/or adaptive changes that arise subsequent to the deformity itself is difficult.

## Melatonin deficiency: pinealectomized animal models

Pinealectomy as a model for scoliosis resembling that seen in humans provides a system to investigate AIS experimentally. Historically, the first evidence of development of scoliosis by pinealectomy and/or damage to the diencephalon was provided by the work of Dubousset et al. [7] in the late 1970s and early 1980s. This area of research remained dormant for almost a decade before being revitalized by the work of Machida et al. [8–13]. As a summary, this group has demonstrated that scoliotic deformity can be consistently produced in chicken by pinealectomy if the surgery is performed shortly after hatching. In fact, pinealectomy resulted in a 100% rate of deformity in all experiments if animals were rendered free of melatonin, the major product of the pineal gland [4, 5, 14, 15]. Furthermore, these investigators demonstrated that development of scoliosis could be prevented by the re-implantation of the pineal gland in skeletal muscle or by the administration of melatonin as a replacement therapy [12]. In addition to chicks, the scoliotic deformity can be produced in pinealectomized rats as well (100% rate of deformity), provided that they were forced to attain a bipedal posture by amputation of the forelimbs and tails [16].

In line with these experimental findings, Machida et al. [17] have also demonstrated that children with progressive AIS had significantly lower levels of blood melatonin when compared to normal controls or those that have non-progressive AIS.

While serotonin (a precursor of melatonin) administration by itself is not very influential in the prevention of deformity, probably because serotonin cannot cross the blood brain barrier, 5-hydroxy-tryptophan (5HT), a serotonin precursor that can cross the blood brain barrier, has been shown to be effective in preventing the development of scoliosis [13]. In further studies, Machida et al. [18] also demonstrated that scoliosis would develop in a genetically melatonin-deficient strain of mice (C57Bl6) again at a rate of 100% when those animals were made bipedal similar to the rat model. Thus, these results indicate that the 5HT-serotonin-melatonin pathway appears to be responsible for the development of scoliosis in this model, and possibly in humans as well.

## Melatonin as a factor in AIS pathogenesis: are the experiments repeatable?

Experimental data by other investigators have failed to echo the findings of Machida et al. [15, 19–22]. Studies of several other groups including Akel et al. have failed to produce scoliotic deformity in pinealectomized chicken with a rate of 100%, even when very low levels of blood melatonin concentrations have been demonstrated. These series reveal consistent rates of scoliotic deformity between 50 and 60% [14, 15, 20–23]. Likewise, rat and C57Bl6 mouse studies performed by us failed to demonstrate uniformity (i.e., 100% rate) in the development of scoliosis [24, 25]. Furthermore, prevention of the appearance of scoliosis by either melatonin administration or pineal gland transplantation in chicken could not be replicated [19, 20]. These results raise the following questions:

1. What is the difference between the work of Machida et al. and other investigations?
2. If very low levels of melatonin are produced in all animals, why do not all animals develop scoliosis?

These questions are unanswered and suggest that other factors may be involved in the pathogenesis of the scoliotic deformity in this model.

Avian species including chicken have a very primitive CNS compared to mammals, and the changes may affect postural mechanisms. Therefore, the inception of a similar model in animals with a more highly evolved CNS is needed. In a study designed to analyze the effects of pinealectomy in mammals (rodents), Bagnall et al. [26] could not produce scoliosis in quadrupedal animals (0% rate). As a result, despite overwhelming evidence provided by Machida et al. [20], and data supporting the fact that the development of scoliosis is not an artifact of the surgical procedure per se, there is still controversy regarding the involvement of the 5HT-serotonin-melatonin pathway in the development of scoliosis in higher animals.

In addition to the controversy among animal models, there is also considerable controversy regarding the blood melatonin levels of human AIS patients and the potential implications of serum melatonin levels on the treatment of AIS. Machida et al. [17] have claimed to demonstrate significantly lower levels of blood melatonin concentration in scoliotic patients with progressive disease which in turn may be reversed by the administration of melatonin, but other studies have disputed the very possibility of detecting low serum melatonin levels in adolescents with AIS [27]. As a summary, it appears that the theory suggesting the very decrease or lack of melatonin in the bloodstreams of affected individuals should be the cause of AIS may be overly simplistic and clinically unproven.

On the other hand, it is almost certain that melatonin and/or another product of the pineal gland may be involved in a number of pathogenetic pathways contributing to the development of AIS. Genetic association studies have demonstrated that a polymorphism in the melatonin receptor 1B (MTNR1B) gene [28] but not melatonin receptor 1A (MTNR1A) gene [29] is associated with the occurrence of AIS. Other studies have demonstrated that there is a defect in melatonin signaling in the osteoblasts of patients with AIS [30] and this defect caused an increased phosphorylation of the serine residues affecting the activity of inhibitory G proteins (G_i_) normally associated with melatonin cell surface receptors [31]. Indeed, Moreau et al. have shown experimentally that all G_i_ (G inhibitory)-coupled receptors are affected in different cell types isolated from AIS patients demonstrating that AIS etiology goes way beyond the classical view that AIS is caused by melatonin deficiency or a pre-existing melatonin signaling defect (Moreau et al, unpublished data, proceedings of the annual meeting of Yves Cotrel Foundation, 2009).

Of note, there is also data that demonstrates abnormalities in the expressions of Ca^2+^ATPases (SERCA; sarco/endoplasmic reticulum Ca^2+^ATPase and PMCA; plasma membrane Ca^2+^ATPase) in platelets as well as in osteoblasts of scoliotic patients, suggesting a defect in cell differentiation involving caspase-3 (i.e., the apoptosis-like phase of the differentiation) [32]. Calmodulin regulates this system. SERCAs pump Ca^2+^ ions into the endoplasmic reticulum (ER), i.e., control the amount of Ca^2+^ into the ER. The ER is a multifunctional organelle supporting many functions and is known to be involved in protein synthesis, translocation across the membrane, integration into the membrane, and posttranslational modification. PMCAs extrude Ca^2+^ from the cytosol and are key molecular components of cellular Ca^2+^ homeostasis and signaling. Changes in Ca^2+^ signaling and handling control muscle development, neuronal precursor differentiation, and osteoblast growth and differentiation [33]. According to this paradigm, melatonin treatment of MEG01 cells (platelet precursor megakaryocytic cell line) results in a dose-dependent increase in Ca^2+^ATPase expression (SERCA3 and PMCA) as well as caspase 3 activity. Therefore, it appears to be an association with melatonin (and/or calmodulin) in the etiopathogenesis of AIS but the actual mechanism(s) of the development of deformity remains unclear.

Work by Acaroglu et al. [34] and other researchers suggest that these interacting mechanisms may well be, but not limited to:

- AIS modeled as a form of neuromuscular scoliosis (in conjunction with an adverse mechanical environment such as bipedality); in which hormonal and other chemical factors act as regulators of skeletal muscle tone and function,
- AIS modeled as a consequence of an abnormality in growth of the spinal column (in conjunction with an adverse mechanical environment such as bipedality); in which hormones and other biochemical factors act as regulators of growth,
- AIS modeled as a mechanical failure of one side of the vertebral column due to a defect in trabecular formation or mineralization (in conjunction with an adverse mechanical environment such as bipedality); in which hormonal and other chemical factors act as regulators of bone formation, mineralization and/or resorption.

## AIS modeled as a form of neuromuscular scoliosis

### Melatonin as a regulator of muscle tone

With the advent of radioactive labeling, melatonin receptors in peripheral tissue can be studied in picomolar affinities and femtomolar densities, revealing their presence in the gastrointestinal system, kidney, lung, heart, vas deferens, and blood vessels [35]. Later research has demonstrated that melatonin was effective in the modulation of vascular smooth muscle tone [36], and intestinal smooth muscle tone [37]. There is also experimental evidence that melatonin and 5-HT4 can affect contractility of chick myocardiocytes in culture [38]. These findings suggest that melatonin or 5-HT4 receptors may play an important role in the modulation of the tone of skeletal muscles as well, which in turn may explain the occurrence of spinal deformity in pinealectomized animals. A review by Pompeiano et al. [39] have suggested a similar association in humans, i.e., the decreased activity of melatonin as well as 5-HT may result in a decrease in the tonus of postural skeletal muscles and may be very important in the pathogenesis of AIS. Alternatively, removal of pineal gland in animals could lead to an increase or decrease in other regulatory protein(s). An analysis of the regulation of melatonin secretion suggests that calmodulin is the neurotransmitter that is effective in turning melatonin secretion on and off [40].

### Calmodulin: what is the significance in AIS?

**Fig. 1:**
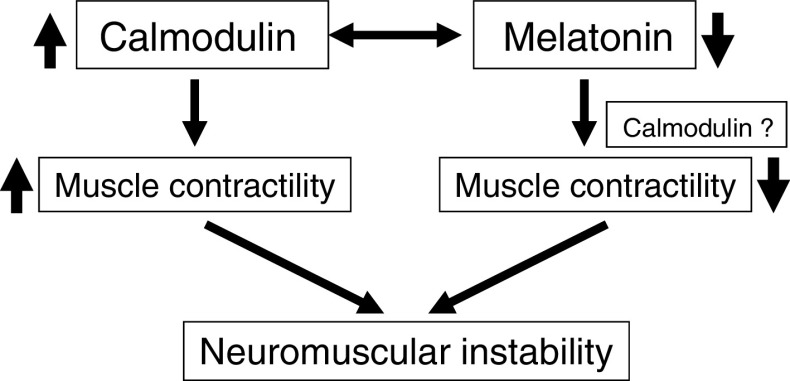
A schematic explanation of the neuromuscular “hypothesis”. Both being shown as muscle tone regulators, any change in the balance between melatonin and calmodulin may effects muscle contractility thereby causing neuromuscular imbalance

Calmodulin is a calcium-binding receptor protein that regulates cAMP-based enzyme systems. Calmodulin regulates the contractile properties of muscle cells by controlling calcium transport through the cell membrane [41], and calmodulin also interacts directly with the contractile proteins, actin and myosin, within the myofiber. Platelet function anomalies were identified in AIS patients long ago [42] but the actual relationship with the spinal pathology still remains to be established. Platelets in patients with AIS have elevated levels of Ca^2+^ and P^i+^ [43, 44], decreased activity of intracellular contractile proteins [43, 45], decreased aggregation [26, 46] and abnormal structure of myosin [43]. Interestingly, several studies have demonstrated that intracellular calmodulin levels are also increased, especially in patients with progressive idiopathic scoliosis [47, 48]. Further studies on platelets performed by Bredoux et al. demonstrated that the expression of Ca^2+^ATPases which regulate the megakaryocytopoiesis (platelet maturation) as well as osteoblast differentiation is abnormal in scoliosis. Further, decreased melatonin levels could account for the abnormality in platelets, because melatonin causes megakaryocyte fragmentation and modulates the cytokine network involved in platelet production [32]. As platelets are thought to resemble “miniature versions of skeletal muscle cells”, these findings suggest that abnormalities in paravertebral muscles of patients with AIS could be primary factors in development and/or progression of spinal deformities. In fact, Zhao et al. [49] had demonstrated that in AIS patients, the expressions of calmodulin and neuronal nitric acid synthase (nNOS) were significantly lower in the paravertebral muscles of the convex side of the deformity. Identification of a relationship between calmodulin activity in platelets and calmodulin activity in paravertebral skeletal muscles may provide a better understanding of mechanisms underlying development and progression of AIS. Hypothetical neuromuscular mechanism caused by the disruption in the balance between melatonin and calmodulin is represented in Fig. 1.

Previous studies by Acaroglu et al. and Akel et al. [24, 34, 50] on the same theme consisted of three different projects to help define the significance of melatonin and calmodulin in the pathogenesis and mechanisms of scoliosis. In the first, tissue levels of melatonin and calmodulin were measured in platelets and skeletal muscle derived from patients with adolescent idiopathic scoliosis undergoing surgical treatment and compared to a group of patients with no underlying chronic spinal problem [34]. Results of this study revealed that contrary to previously published reports, tissue calmodulin levels were not significantly different in scoliotics compared to controls, a finding that indicates platelet calmodulin levels may not be adequate to use as a screening tool for the impending onset of scoliosis. Likewise, tissue melatonin concentrations were not significantly different in scoliotics compared to controls. Based on these findings, it may be postulated that in accordance with the studies demonstrating no differences in serum levels, there are probably no differences in tissue levels as well. Furthermore, it was also demonstrated that skeletal muscle calmodulin content was significantly different when the convex side was compared to the concave side. However, contrary to the findings of Zhao et al. cited above [49], the results of Acaroglu et al. showed an increase of muscle calmodulin levels at the convex side. This is an important finding as it demonstrates that calmodulin may be involved in the regulation of contraction of skeletal muscles and electrophysiological differences between these muscles may reflect differences in calmodulin concentration.

Although calmodulin is probably not a causative factor, this molecule may be one of the important factors contributing to the progression of scoliotic curves. In other words, it may be hypothesized that calmodulin is probably not the factor triggering the occurrence of deformity but the secondary imbalance in calmodulin content may be the factor governing progression [34].

This hypothesis was tested in two other studies using animals models, a pinealectomized chicken model and a C57Bl6 mice model, attempting to antagonize calmodulin using two different CaM antagonist pharmacological agents, tamoxifen (TMX) and trifluoperazine (TFP) [24, 51] (In fact, an earlier study by Enouf et al. [51] has shown that TFP inhibited the Ca^2+^ transport mediated by Ca^2+^ATPases in microsomal platelet fractions). Findings of these studies demonstrated that as hypothesized, calmodulin inhibitors do not prevent the occurrence of scoliotic deformities in either the pinealectomized chicken or the C57BL6 mice models. On the other hand, TMX was shown to decrease the rate of progression of deformity in both models. This observation suggests that the hypothesis on the influence of calmodulin on the progression of the deformity may be correct. In addition, in both models, tamoxifen with or without the addition of trifluoperazine led to the reversal of curvature in a significantly higher number of animals compared to controls. Interestingly, TMX and raloxifen (RLX) appear to have a negative effect on the smooth muscle tone of vascular walls as well [52] and their mechanism of action in the reversal of deformity in animal models may be based on this effect. However, it has to be stressed that the action of TMX might not have necessarily been based on its’ affect on calmodulin but by way of a different interaction, specifically the regulatory effect on estrogen or estrogen regulated proteins.

## AIS modeled as a local defect in the growth of the spinal column

The natural history of adolescent idiopathic scoliosis is strongly associated with growth. Clinically, it can easily be claimed that this is an ascending (increasing/growing) type of scoliosis (as opposed to the descending/collapsing types such as neuromuscular or degenerative) as the initiation of the deformity is during a period of very rapid growth. Similarly, progression of spinal curves is closely interrelated with the actual rate of growth, as well as, with the remaining potential of growth in affected individuals. This phenomenon of dependency on growth is also evident in the animal models discussed above, because there is no evidence suggesting that experimental models of scoliosis can also be developed in mature animals.

It is a common knowledge that certain types of spinal deformity such as “congenital deformity” arise from the growth imbalance within the vertebral column; emerging as scoliosis if the asymmetry is predominantly in the coronal plane, as kyphosis or more rarely as lordosis if in the sagittal plane; or as kyphoscoliosis in case it is in both planes. It is plausible that similar growth plate abnormalities may be present in patients with idiopathic scoliosis as well. An MRI study by Day et al. [53] suggest abnormalities albeit similar at the concave and convex sides of the growth plates, suggesting a primary disturbance of growth as a potential cause for the development of deformity. Likewise, Rusova et al. [54] demonstrated a significant decrease in sulfation and acetylation of glycosaminoglycans suggesting a primary defect in the function of cells in the growth plates. However, histomorphological studies of vertebral endplates obtained from animal models of scoliosis as well as human subjects have only demonstrated evidence suggesting that uneven loading leads to differences in cell proliferation [55], cell differentiation [44] and changes in collagen composition [56], but they failed to show any hint of a primary disturbance of growth.

Another possible mechanism in which a problem of growth may be associated could be a defect in the modulation of growth in patients with AIS. Indeed, genetic studies suggest several single nucleotide polymorphisms (SNPs) in genes coding proteins that regulate growth may be associated with the development or progression of scoliosis in AIS [57–59]. These SNPs may also be associated with the generalized osteopenia in AIS patients [57] (see discussion below). Qiu et al. [60] demonstrated a significant decrease in circulating leptin levels in patients with AIS, which is associated with altered growth parameters and changes in bone mineral density/content (BMD/BMC). More recently, Liu et al. [61] measured serum leptin and sOB-R concentrations by enzyme-linked immunosorbent assay (ELISA) and showed that AIS girls were found to have significantly higher sOB-R level and lower free leptin index (FLI) after adjusting for age and body weight in multivariate regression analysis. Finally, unpublished data by Moreau et al. suggest that increased activity of certain cytokines may be associated with the development and progression of scoliosis in melatonin deficiency animals (pinealectomized chicken and C57BL6 mice), as well as, in AIS patients (Moreau, unpublished data, proceedings of the annual meeting of Yves Cotrel Foundation, 2009). In conclusion, there is evidence suggesting the presence of a growth disturbance in patients with AIS, but this domain remains to be the least explored of the hypothetical mechanisms listed above.

## AIS modeled as a mechanical failure of one side of the vertebral column due to a defect in trabecular formation or mineralization

The clinical association of AIS with osteopenia was brought to attention by the work of Cheng et al. [62–65]. Although it was assumed that this finding might be the consequence of a problem in vitamin D synthesis or metabolism, no defects in this system or appropriate genetic polymorphisms could be identified [66, 67]. Unpublished pilot data by Acaroglu et al. on the bipedal C57Bl6 model also suggests that the animals with scoliosis have significantly less trabecular density compared to those animals that received TMX treatment. In an effort to replace TMX with a more specific selective estrogen receptor modulator (SERM) RLX, this group demonstrated that RLX was as effective as TMX in inducing regression of scoliotic curves in the mouse model, and also that administration of pharmacological agents after the development of scoliosis (i.e., the 20th week in mice model) might be almost as effective as the preemptive use.

Based on these observations another study was performed in which osteoporosis was investigated as the primary factor in the development of experimental scoliosis. In this study, we compared the frontal and sagittal spinal alignment in bipedal rats rendered osteoporotic with subcutaneous heparin injections compared to controls. Results of this study [25] failed to demonstrate any significant differences in rates of scoliosis, nor of curve magnitudes.

However, it was very interesting to see that the majority of non-melatonin-deficient bipedal animals did develop deformity, more so when they were osteoporotic. Another significant finding of this study was a significant decrease in the amount of kyphosis in the osteoporotic group, which is consistent with the clinical observations in human patients with AIS. This finding is unique in the sense that the sagittal plane had not been evaluated in the previous studies and may be very important in our understanding of the three dimensional scoliotic deformity in these models. However, these findings do suggest that SERM, such as TMX and RLX may be effective in the reversal of osteopenia and scoliotic deformity in animal models. In other words, although the experiments with these molecules were started with the assumption that their CaM antagonism had been the mechanism of action, results suggest that estrogen receptor modulation may be the key factor in the observed effects; especially considering that RLX probably does not have any anti-CaM properties.

These observations suggest that estrogen and/or estrogen receptors may be key factors in the pathogenesis of AIS. This assumption indeed makes clinical sense as it may explain the female predilection associated with the disease. Furthermore, it may also explain the observation of osteopenia in scoliotic individuals, potentially similar to the osteopenia observed following menopause. Estrogen receptor gene polymorphism has been recognized to be associated with AIS in humans in work by Inoue and by Moldovan et al. [67]. Research by Dr Moldovan’s laboratory has shown that estrogens play a critical role in AIS [68, 69] through their impact on bone cell signaling and function. Their results indicate that estrogens are not at the origin of AIS; however, they interact with the osteoblast signaling defect in AIS patients. Indeed, estrogens are known to repress and reduce the synthesis of G proteins both at the transcriptional and translational levels. In this respect, Moreau et al. have proposed a model explaining the incidence of AIS around puberty and why girls are more affected in number and severity due to a cross-talk between estrogens and the pre-existing melatonin signaling defect in AIS [70]. Moldovan et al. [31, 70] has previously demonstrated that 17-β-estradiol can reduce cAMP production in a specific subgroup of AIS patients identified according to the functional classification of Moreau et al. [31, 70], i.e., melatonin-responsive AIS patients. In this work, the molecular mechanism whereby this reduction occurs was characterized. The melatonin receptor MT2, which is normally physiologically coupled with the G_i_ protein, switches to the G_s_ protein in the osteoblasts of a specific group of AIS patients when their cells are exposed to 17-β-estradiol. Effect of estrogens on osteoblast metabolism is well documented in the literature and in clinical practice, but curiously, little was known regarding AIS. Osteoblast differentiation impacts the rigidity, elasticity, and mechanical properties of bone, involving an increase in alkaline phosphatase (ALP) activity, extracellular matrix (ECM) synthesis, and bone mineralization, and an existing defect of this was also observed. It is well known that estrogens promote high AP activity, collagen synthesis and calcium deposition in bone ECM. In these studies, it was verified the osteoblastic phenotype of cells derived from bone biopsies, but the effect of 17-β-estradiol on osteoblast gene markers for differentiation (osteocalcin, osteopontin, BSP, and ALP activity) could not be demonstrated. However, these effects have been clearly established in many studies, and it is widely accepted that estrogen depletion reduces osteoblast activity. This impacts the balance between the resorption and formation of bone, and can lead to osteopenia present in AIS patients as discussed above.

## The common denominator: bipedality

Clinical observations and animal models suggest that the common denominator in the occurrence of scoliosis may be bipedality. To date, scoliosis can be neither experimentally produced nor observed naturally in any quadrupedal animal. Our previous work discussed above also suggests that bipedality is essential for the production of scoliosis [25]. In 2005, Castelein et al. [71] offered a possible explanation of the association between bipedality and the development of scoliosis. These investigators suggest a shift of the moments acting on the spinal column from being purely flexor in the quadrupedal animals to being dominantly extensor in humans standing upright [71–73]. This extensor moment may unlock the facet joints that are very effective in resisting the anterior translation but not so in resisting the posterior translation of one vertebra over the other. The resulting instability may in turn produce a rotational deformation of the involved functional unit, triggering the onset of scoliotic deformity [73]. This theory also explains the fact that AIS is almost unique to humans as the bipedality of other primate species is not associated with the same upright posture. In fact, apart from humans there is only one case report of an orangutan with scoliosis that was attributed to a probable neuromuscular etiology secondary to a CNS infection [74].

Of note, it has recently been postulated that scoliosis may also be initiated in several fish species including salmon [75] and a wider group of teleosts [76] after pinealectomy. In their study, Fjelldal et al. [75] have demonstrated that the development of scoliosis in pinealectomized salmon was associated with a decreased BMC, decreased stiffness, yield limit and resilience of the vertebral bodies which may in turn yield to the massive body musculature of the fish. This finding is important because it demonstrates that pinealectomy in fish also causes osteopenia and related mechanical problems of the spine. In addition, the massive body musculature of the fish might be exerting an axial force comparable to gravity in humans (i.e., fish models of spinal loading might be more appropriate than quadruped animal models).

**Fig. 2:**
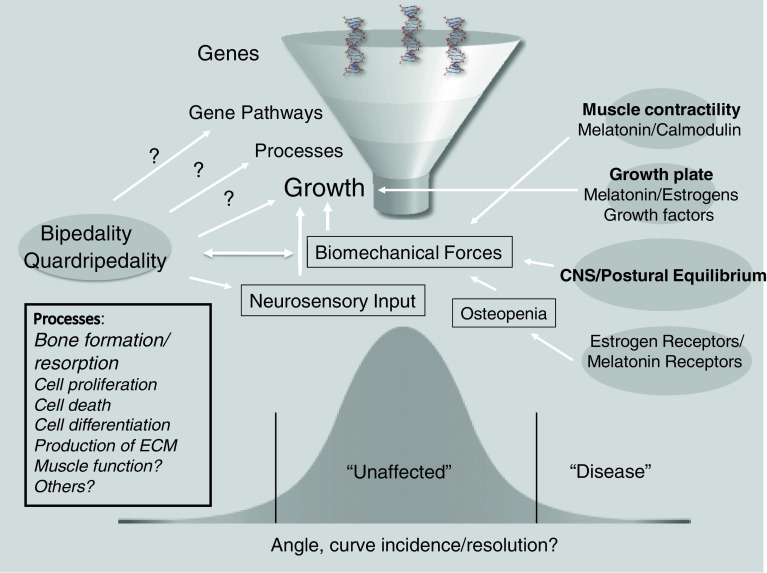
A schematic explanation of the interaction between genetic, metabolic and mechanical (postural) factors in the development of scoliosis. Please note that (1) scoliosis may follow various mechanistic pathways to develop in different individuals and (2) the genetical background of these pathways may be present in the majority of the general population while only outliers (i.e., individuals with several factors) are affected

The effects of bipedality are probably not limited to deformities in the coronal plane of the spinal column but other planes have not been investigated in animal models to date. In addition, possible structural changes in the pelvis and spinopelvic junction in spinal columns with or without any scoliotic deformity are virtually unknown. It is reasonably probable that especially in the experimental animal models, the initiation of scoliotic deformity may be secondary to an extensor or rotational moment that may trigger the scoliotic deformity in susceptible animals as a consequence of a metabolic or hormonal disorder (as outlined above). For this reason, it may be very important to analyze the metabolic and/or endocrine factors in conjunction with the postural factors in the absence of which no deformity would develop (Fig. 2).

## Future work

Analysis of the structural as well as biochemical changes in the spinal columns of experimental bipedal animals would also shed light on the origins of degenerative problems of the spinal column in humans. These studies may answer some of the following fundamental questions:

1. Do the findings in animal models reflect similar pathologies in humans, and likewise, do the findings in animal models reflect pathologies in humans?
2. Assuming that pinealectomy or other anti-melatonin interventions produce similar biochemical and hormonal consequences, why is it that all bipedal melatonin-deficient animals do not produce scoliosis? The explanation for this may be mechanical or postural and might be identified with the analysis of the three-dimensional spinopelvic parameters of these animals, or may be based on the fact that our assumption above is indeed wrong.
3. Do the growth and/or mechanical problems (i.e., osteopenia) associated with scoliosis develop before or secondary to the occurrence of scoliotic deformity? With the possibility of analyzing the spinal columns in 3D at the time of histomorphometric analysis, the triggering event that produces growth and/or mechanical disturbance as well as that producing the deformity may be identified.
4. Which pharmacological agents (TMX, RLX, estrogen, NO donors) administered may be effective in arresting and/or reversing the deformity? At which time point(s) are they effective, and upon which mechanism do they act?
5. With the understanding that we are not at the point of identifying the exact etiology of AIS, could we possibly identify the pathological mechanisms effective in the occurrence and progression of the deformity, and based on this, could we treat AIS in humans with pharmacological agents?
6. Finally, if the mechanisms associated with occurrence and progression is predominantly mechanical in some individuals (animals) and biochemical in others, can we identify these subsets in order to treat one group with mechanical measures (this group may be more amenable to brace treatment), and the other with hormonal/pharmacological measures (assuming this group will be more resistant to brace treatment)?

## References

[1] Ahn UM, Ahn NU, Nallamshetty L (2002). The etiology of adolescent idiopathic scoliosis. Am J Orthop.

[2] Lowe TG, Edgar M, Margulies JY (2000). Etiology of idiopathic scoliosis: current trends in research. J Bone Joint Surg Am.

[3] Miller NH (1999). Cause and natural history of adolescent idiopathic scoliosis. Orthop Clin North Am.

[4] Porter RW (2001). The pathogenesis of idiopathic scoliosis: uncoupled neuro-osseous growth?. Eur Spine J.

[5] Roach JW (1999). Adolescent idiopathic scoliosis. Orthop Clin North Am.

[6] Miller NH (2002). Genetics of familial idiopathic scoliosis. Clin Orthop.

[7] Dubousset J (1983). Experimental scoliosis induced by pineal and diencephalic lesions in young chickens: its relation with clinical findings. Orthop Trans.

[8] Machida M (1999). Cause of idiopathic scoliosis. Spine.

[9] Machida M, Dubousset J, Imamura Y (1995). Role of melatonin deficiency in the development of scoliosis in pinealectomized chickens. J Bone Joint Surg Br.

[10] Machida M, Dubousset J, Imamura Y (1994). Pathogenesis of idiopathic scoliosis: SEPs in chicken with experimentally induced scoliosis and in patients with idiopathic scoliosis. J Pediatr Orthop.

[11] Machida M, Dubousset J, Imamura Y (1996). Melatonin. A possible role in pathogenesis of adolescent idiopathic scoliosis. Spine.

[12] Machida M, Dubousset J, Satoh T (2001). Pathologic mechanism of experimental scoliosis in pinealectomized chickens. Spine.

[13] Machida M, Miyashita Y, Murai I (1997). Role of serotonin for scoliotic deformity in pinealectomized chicken. Spine.

[14] Turgut M, Yenisey C, Uysal A (2003). The effects of pineal gland transplantation on the production of spinal deformity and serum melatonin level following pinealectomy in the chicken. Eur Spine J.

[15] Turhan E, Acaroglu E, Bozkurt G (2006). Unilateral enucleation affects the laterality but not the incidence of scoliosis in pinealectomized chicken. Spine.

[16] Machida M, Murai I, Miyashita Y (1999). Pathogenesis of idiopathic scoliosis. Experimental study in rats. Spine.

[17] Machida M, Dubousset J, Yamada T, Kimura J (2009). Serum melatonin levels in adolescent idiopathic scoliosis prediction and prevention for curve progression: a prospective study. J Pineal Res.

[18] Machida M, Dubousset J, Yamada T (2006). Experimental scoliosis in melatonin-deficient C57BL/6 J mice without pinealectomy. J Pineal Res.

[19] Bagnall K, Raso VJ, Moreau M (1999). The effects of melatonin therapy on the development of scoliosis after pinealectomy in the chicken. J Bone Joint Surg Am.

[20] Bagnall KM, Beuerlein M, Johnson P (2001). Pineal transplantation after pinealectomy in young chickens has no effect on the development of scoliosis. Spine.

[21] Wang X, Jiang H, Raso J (1997). Characterization of the scoliosis that develops after pinealectomy in the chicken and comparison with adolescent idiopathic scoliosis in humans. Spine.

[22] Wang X, Moreau M, Raso VJ (1998). Changes in serum melatonin levels in response to pinealectomy in the chicken and its correlation with development of scoliosis. Spine.

[23] Buerlein M, Wang X, Moreau M, Raso J, Mahood J, Bagnall K (2001). Development of scoliosis after pinealectomy in young chickens is not the result of an artifact of the surgical procedure. Microsc Res Tech.

[24] Akel I, Demirkiran G, Alanay A (2009). The effect of calmodulin antagonists on scoliosis: bipedal C57BL/6 mice model. Eur Spine J.

[25] Dede O, Akel I, Demirkiran G, et al (2011). Is decreased bone mineral density associated with development of scoliosis? a bipedal osteopenic rat model. Scoliosis (in review) 10.1186/1748-7161-6-24 PMC3217908 22040734

[26] Floman Y, Liebergall M, Robin GC (1983). Abnormalities of aggregation, tromboxane A2 synthesis, and C14 serotonin release in platelets of patients with idiopathic scoliosis. Spine.

[27] Bagnall K, Raso VJ, Hill DL (1996). Melatonin levels in idiopathic scoliosis. Diurnal and nocturnal serum melatonin levels in girls with AIS. Spine.

[28] Qiu XS, Tang NL, Yeung HY (2007). Melatonin receptor 1B (MTNR1B) gene polymorphism is associated with the occurrence of adolescent idiopathic scoliosis. Spine.

[29] Qiu XS, Tang NL, Yeung HY (2008). Lack of association between the promoter polymorphism of the MTNR1A gene and adolescent idiopathic scoliosis. Spine.

[30] Moreau A, Wang DS, Forget S (2004). Melatonin signaling dysfunction in adolescent idiopathic scoliosis. Spine.

[31] Azeddine B, Letellier K, da Wang S, Moldovan F, Moreau A (2007). Molecular determinants of melatonin signaling dysfunction in adolescent idiopathic scoliosis. Clin Orthop Relat Res.

[32] Bredoux R, Corvazier E, Dally S (2006). Human platelet Ca2 + -ATPases: new markers of cell differentiation as illustrated in idiopathic scoliosis. Platelets.

[33] Zayzafoon M (2006). Calcium/calmodulin signaling controls osteoblast growth and differentiation. J Cell Biochem.

[34] Acaroglu E, Akel I, Alanay A, Yazici M, Marcucio R (2009). Comparison of the melatonin and calmodulin in paravertebral muscle and platelets of patients with or without adolescent idiopathic scoliosis. Spine.

[35] Pang SF, Dubucovich ML, Brown GM (1993). Melatonin receptors in peripheral tissues: a new area of melatonin research. Biol Signals.

[36] Mahle CD, Goggins GD, Agarwal P (1997). Melatonin modulates vascular smooth muscle tone. J Biol Rhythms.

[37] Luchelli A, Santagostino-Barbone MG, Tonini M (1997). Investigation into the contractile response of melatonin in the guinea pig isolated proximal colon: the role of 5-HT4 and melatonin receptors. Br J Pharmacol.

[38] Mei YA, Lee PP, Wei H (2001). Melatonin and its analogs potentiate the nifedipine sensitive high-voltage-activated calcium current in the chick embriyonic heart cells. J Pineal Res.

[39] Pompeiano O, Manzoni D, Miele F (2002). Pineal gland hormone and idiopathic scoliosis: possible effect of melatonin on sleep-related postural mechanisms. Arch Ital Biol.

[40] Xia Z, Storm DR (1997). Calmodulin-regulated adenylyl cyclases and neuromodulation. Curr Opin Neurobiol.

[41] Cheung WY (1980). Calmodulin plays a pivotal role in cellular regulation. Science.

[42] Yarom R, Meyer S, More R (1982). Metal impregnation abnormalities in platelets of patients with idiopathic scoliosis. Haemostasis.

[43] Peleg I, Eldor A, Kahane I (1989). Altered structural and functional properties of myosins from platelets of idiopathic scoliosis patients. J Orthop Res.

[44] Yarom R, Blatt J, Gorodetsky R (1980). Microanalysis and X-ray fluorescence spectrometry of platelets in diseases with elevated muscle calcium. Eur J Clin Invest.

[45] Muhlrad A, Yarom R (1982). Contractile proteins in platelets from patients with idiopathic scoliosis. Haemostasis.

[46] Sabato S, Rotman A, Robin GC (1985). Platelet aggregation abnormalities in idiopathic scoliosis. J Pediat Orthop.

[47] Kindsfater K, Lowe TG, Lawellin D (1994). Levels of platelet calmodulin for the prediction of progression and severity of adolescent idiopathic scoliosis. J Bone Joint Surg Am.

[48] Lowe T, Lawellin D, Smith D (2002). Platelet calmodulin levels in adolescent idiopathic scoliosis. Spine.

[49] Zhao Y, Qiu GX (2004). Expression of calmodulin and nNOS in the paraspinal muscles in idiopathic Scoliosis. Zhonghua Yi Xue Za Zhi.

[50] Akel I, Kocak O, Bozkurt G (2009). The effect of calmodulin antagonists on experimental scoliosis: a pinealectomized chicken model. Spine.

[51] Enouf J, Lévy-Toledano S (1984). Relationship between structure of phenothiazine analogues and their activity on platelet calcium fluxes. Br J Pharmacol.

[52] Leung FP, Tsang SY, Wong CM (2007). Raloxifene, tamoxifen and vascular tone. Clin Exp Pharmacol Physiol.

[53] Day G, Frawley K, Phillips G (2008). The vertebral body growth plate in scoliosis: a primary disturbance of growth?. Scoliosis.

[54] Rusova TV, Rykova VI, Korel AV, Zaidman AM, Tkachev DS (2005). Glycosaminoglycans of the vertebral body growth plate in patients with idiopathic scoliosis. Bull Exp Biol Med.

[55] Yoshihara H, Kawakami N, Matsuyama Y (2005). A histomorphologic study of scoliosis in pinealectomized chickens. Spine.

[56] Cancel M, Grimard G, Thuillard-Crisinel D, Moldovan F, Villemure I (2009). Effects of in vivo static compressive loading on aggrecan and type II and X collagens in the rat growth plate extracellular matrix. Bone.

[57] Il-Soo E, Weon WK, Kuen TS, Jeung IK, Jung SL (2009). Association between osteoprotegerin gene polymorphism and bone mineral density in patients with adolescent idiopathic scoliosis. Eur Spine J.

[58] Yang Y, Wu Z, Zhao T (2009). Adolescent idiopathic scoliosis and the single-nucleotide polymorphism of the growth hormone receptor and IGF-1 genes. Orthopedics.

[59] Yeung HY, Tang NL, Lee KM (2006). Genetic association study of insulin-like growth factor-I (IGF-I) gene with curve severity and osteopenia in adolescent idiopathic scoliosis. Stud Health Technol Inform.

[60] Qiu Y, Sun X, Qiu X (2007). Decreased circulating leptin level and its association with body and bone mass in girls with adolescent idiopathic scoliosis. Spine.

[61] Liu Z, Tam EM, Sun GQ, et al (2011) Abnormal leptin bioavailability in adolescent idiopathic scoliosis: an important new finding. Spine 36 (Epub ahead of print) 10.1097/BRS.0b013e318227dd0c 21681139

[62] Cheng JC, Guo X (1997). Osteopenia in adolescent idiopathic scoliosis. A primary problem or secondary to the spinal deformity?. Spine.

[63] Cheng JC, Guo X, Sher AH (1999). Persistent osteopenia in adolescent idiopathic scoliosis. A longitudinal follow up study. Spine.

[64] Cheng JC, Qin L, Cheung CS (2000). Generalized low areal and volumetric bone mineral density in adolescent idiopathic scoliosis. J Bone Miner Res.

[65] Cheng JC, Tang SP, Guo X, Chan CW, Qin L (2001). Osteopenia in adolescent idiopathic scoliosis: a histomorphometric study. Spine.

[66] Chen WJ, Qiu Y, Zhu F (2008). Vitamin D receptor gene polymorphisms: no association with low bone mineral density in adolescent idiopathic scoliosis girls. Zhonghua Wai Ke Za Zhi.

[67] Inoue M, Minami S, Nakata Y (2002). Association between estrogen receptor gene polymorphisms and curve severity of idiopathic scoliosis. Spine.

[68] Leboeuf D, Letellier K, Alos N, Edery P, Moldovan F (2009). Do estrogens impact adolescent idiopathic scoliosis?. Trends Endocrinol Metab.

[69] Letellier K, Azeddine B, Parent S (2008). Estrogen cross-talk with the melatonin signaling pathway in human osteoblasts derived from adolescent idiopathic scoliosis patients. J Pineal Res.

[70] Moreau A, Akoumé Ndong MY, Azeddine B (2009). Molecular and genetic aspects of idiopathic scoliosis. Blood test for idiopathic scoliosis. Orthopade.

[71] Castelein RM, van Dieën JH, Smit TH (2005). The role of dorsal shear forces in the pathogenesis of adolescent idiopathic scoliosis: a hypothesis. Med Hypotheses.

[72] Kouwenhoven JW, Smit TH, van der Veen AJ (2007). Effects of dorsal versus ventral shear loads on the rotational stability of the thoracic spine: a biomechanical porcine and human cadaveric study. Spine.

[73] Kouwenhoven JW, Castelein RM (2008). The pathogenesis of adolescent idiopathic scoliosis: review of the literature. Spine.

[74] Naique SB, Porter R, Cunningham AA (2003). Scoliosis in an orangutan. Spine.

[75] Fjelldal PG, Grotmol S, Kryvi H (2004). Pinealectomy induces malformation of the spine and reduces the mechanical strength of the vertebrae in Atlantic salmon, Salmo salar. J Pineal Res.

[76] Gorman KF, Breden F (2009). Idiopathic-type scoliosis is not exclusive to bipedalism. Med Hypotheses.

